# Beginning your hearing health journey with the online hearing test

**DOI:** 10.3399/BJGPO.2023.0021

**Published:** 2023-06-28

**Authors:** Crystal Rolfe, Ayla Ozmen, Devina Maru

**Affiliations:** 1 Associate Director of Health, Royal National Institute for Deaf People, London, UK; 2 Expertise and Policy Lead, Royal National Institute for Deaf People, London, UK; 3 Primary Care Doctor, Royal College of General Practitioners Honorary Clinical Advisor and Deafness and Hearing Loss Toolkit Lead, London, UK; 4 NIHR UCLH Biomedical Research Centre Deafness and Hearing Problems Theme Board Member, London, UK

**Keywords:** ENT, Health promotion and prevention, Screening, Audiology, General practice, Primary healthcare

## Introduction

There are 12 million people with hearing loss in the UK and this is expected to increase to 14.2 million by 2035. We know that the effects of unmanaged hearing loss are significant. It leads to an increased risk of both social isolation and poor mental health, and is a major driver for leaving the workforce early. Hearing loss has also been identified as the largest potentially modifiable risk factor for dementia. Yet still, people wait an average of 10 years before they seek help for their hearing loss. World Hearing Day 2023^1^ took place on the 3 March and the theme this year looks to highlight the importance of integrating ear and hearing care within primary care, as an essential component of universal health coverage.

### Beginning your hearing health journey with the online hearing test

In 2022, the Royal National Institute for Deaf People (RNID), celebrated over 100 000 people taking their free online hearing check^2^ and beginning their hearing health journey. The check takes just 3 minutes and measures how well you can hear speech in background noise. Those whose result shows that they may have hearing loss are signposted to their GP. In line with The National Institute for Health and Care Excellence (NICE) guidance, RNID recommends GPs investigate this potential hearing loss by taking the actions listed in Box 1.

Box 1 **NICE Guidance [NG98]**

The following is recommended:

- otoscopy to exclude impacted wax or infection, then
- arrange an audiological assessment, and
- refer for additional diagnostic assessment if needed.

### The nation’s hearing health

We know that hearing loss is a growing public health challenge in the UK, and RNID have released research which painted a startling picture of the nation’s hearing health.^3^

Despite 91% of adults saying they rate their hearing as good, the research found that:

- more than a third (34%) admit they’ve pretended to follow conversations they've struggled to hear, for example by smiling and nodding along;
- the same amount (34%) have found it difficult to follow conversations in noisy surroundings like restaurants or parties;
- almost a quarter (24%) have found themselves asking people to repeat themselves;
- more than 1 in 10 (13%) say they have been told by their partner they think they might have a problem with their hearing;
- however, 83% have never taken any action in relation to their hearing, although 74% said they would be likely to take an online hearing check that is free and takes 3 minutes.

### The benefits of hearing aids and early intervention

It is well established that hearing aids have a large beneficial effect on people’s ability to take part in everyday life. NICE recommends hearing aids as the most cost-effective intervention for hearing loss.^4^ There is also a growing body of evidence to suggest that hearing aids slow cognitive decline.^5^ This evidence has become particularly pertinent in the light of the 2020 Lancet Commission on dementia, which identified hearing loss as the largest potentially modifiable risk factor for dementia, with unmanaged hearing loss responsible for up to 8.2% of all dementia cases.^6^

Yet many people experiencing hearing loss do not seek medical advice and remain undiagnosed. Typically, people who are referred for a hearing assessment are aged in their mid-70s and on average wait 10 years from the initial onset of hearing loss until they seek medical advice.^7^ It is estimated that in England, only 2 million people have hearing aids out of the 6 million who have hearing loss that is significant enough to benefit from them.^7^

The reasons for delaying seeking medical advice may be due to multiple factors. Individuals may underestimate the serious effects that hearing loss can have; they may fear the stigma associated with hearing loss and hearing aids; they may not realise the impact it is having due to its gradual progression; they may be in denial; or they may simply deprioritise it.^7^

We know that the ability to adapt to and manage hearing loss becomes increasingly difficult the older people are when they make an appointment for assessment and intervention.^8^ We also know that hearing loss becomes a risk factor for dementia in mid-life.^6^ Supporting earlier identification and intervention, therefore, could ensure that individuals are supported to manage their hearing loss at an age when they are likely to benefit the most.

For this reason, NICE recommends referring adults with diagnosed or suspected dementia, or mild cognitive impairment for hearing assessment in audiology. NICE also recommends referring these patients and patients with a diagnosed learning disability for hearing assessment every 2 years.

### Top tips for GPs

GPs have the power to improve their patients’ hearing health with a potentially transformative effect on people’s overall health and quality of life, through preventing social isolation, supporting mental health, enabling work, and potentially slowing cognitive decline. RNID has set out some clear guidance to help GPs identify and support their patients with hearing loss to access the management that they need.

- If someone has taken the RNID hearing test and had an outcome that suggests that they could have hearing loss, refer them for a full diagnostic test with a specialist as shown in Box 1. There are free NHS audiology services available to all adults over the age of 18 in every area across the UK, where these services can be found will differ according to local area.
- Over 70% of over 70 year olds have hearing loss, with a high proportion undiagnosed. If you notice a patient struggling to hear you, discuss this with them and offer to refer them for a full diagnostic test with a specialist.
- Share the RNID free online hearing test.^2^ It takes three minutes and can be done in the comfort of one's own home or at the GP surgery in a quiet room. It isn’t the same as a full hearing test, but it’s a reliable way to find out if your patient needs one.
- RNID collaborated with the Royal College of General Practitioners and NHS England in developing a toolkit^9^ to educate GPs on D/deafness and hearing loss. Find the D/deafness and hearing loss toolkit here.
- Ensure GP services are accessible to people with hearing loss and comply with the Accessible Information Standard in England. Practical tips on accessibility and communicating with patients who are D/deaf or have hearing loss are available via the RNID website.
- RNID has recently called for ear wax removal services to be brought back into primary care or community settings. If one of your patients is suffering from a build-up of ear wax, refer them to a local NHS removal service.

### Further information

The RNID hearing check is suitable for most adults, however the check should not be recommended if any of the following apply:

- unilateral or asymmetric hearing loss (and pain, wax, or infection has been excluded);
- hyperacusis or severe tinnitus;
- suspected conductive hearing loss;
- diagnosed or suspected dementia or cognitive impairment;
- diagnosed learning disabilities; or
- under 18 years old.

Instead a referral for diagnostic assessment in ENT or audiology should be made, in line with NICE guidelines and local pathways.
